# Supplementary material for: Transcription Factor CcFoxO Mediated the Transition from Summer Form to Winter Form in Cacopsylla chinensis
Source: Int J Mol Sci. 2024 Aug 5;25(15):8545. doi: 10.3390/ijms25158545 (PMC11313232; doi:10.3390/ijms25158545)
Supplement: Supplementary file 1 [file ijms-25-08545-s001.zip › ijms-3123528-supplementary.pdf]

Supplementary Materials

Article

Transcription Factor *CcFoxO* Mediated the Transition from Summer Form to Winter Form in *Cacopsylla chinensis*

Chuchu Wei [1] and Songdou Zhang [1,2,*]

Table S1. List of primers used in this study.

Gene name and GenBank ID	Forward Primer (5'-3')	Reverse Primer (5'-3')	Product size (bp)
CcFoxO (PP931994)	Full-F: ATGGATCAACTCGTTTTCAA	Full-R: TTAGTGAACCCAGGATTGTC	1296
	qF: CAAGTTCTCAGCACACTCAA	qR: GGTCTCCCTCTAACGAGATT	127
	RNAi-F: ggatcctaatacgactcactatagg TCTGATCTGGACGTAGACTT	RNAi-F: ggatcctaatacgactcactatagg CCGGAGATGAAGTAATAGCC	351
CcTRPM [18] (OQ658558)	qF: ACAATGCATTCTTCTTGACC	qR: GACTTTGTACACTGGGGGTA	141
	RNAi-F: ggatcctaatacgactcactatagg GGTACCTTCGTATCCTCAAC	RNAi-F: ggatcctaatacgactcactatagg TGGCATTGATCTCCATGAAA	472
EGFP (ACY56286)	RNAi-F: ggatcctaatacgactcactatagg ACTCCAGCAGGACCATGTGATC	RNAi-F: ggatcctaatacgactcactatagg CCTGAAGTTCATCTGCACCAC	596
Ccβ-actin (OQ658571)	qF: CGTATGCAGAAGGAAATCAC	qR: AGATCCACATCTGTTGGAAG	139

Note: The T7 promoter sequences in the primers of dsRNA synthesis are shown in lowercase fashion.

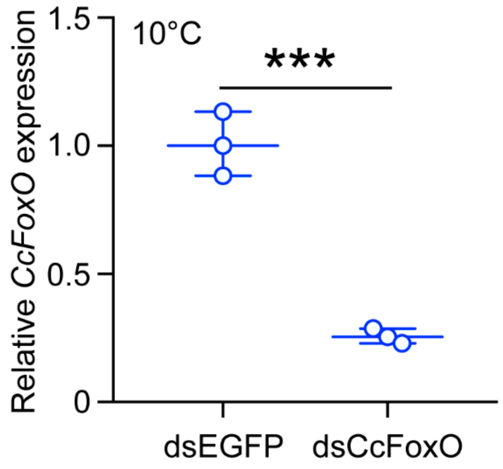

Figure S1. RNAi efficiency of *CcFoxO*. The mRNA expression of *CcFoxO* in SF 1st instar nymphs treated with dsEGPF and dsCcFoxO at 15 days under 10 °C(n=3). Data are presented as mean ± SD. Each circle represents a set of repetitions. Statistically significant differences were determined with the pair-wise Student's *t*-test, and significance levels were denoted by *** (p<0.001).

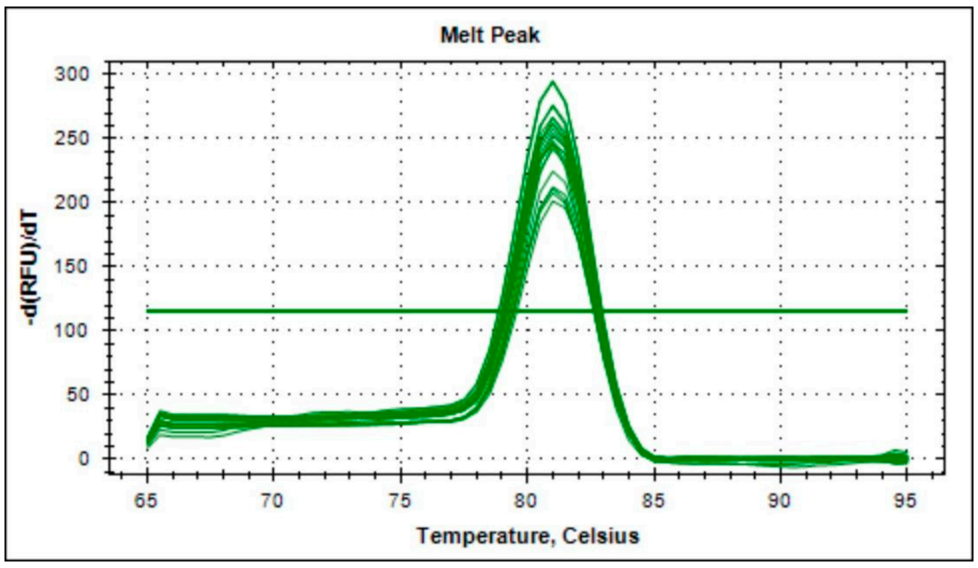

Figure S2. The melting curves for qRT-PCR primers of *CcFoxO*.
